# Multi-part and scale adaptive visual tracker based on kernel correlation filter

**DOI:** 10.1371/journal.pone.0231087

**Published:** 2020-04-13

**Authors:** Mingqi Luo, Bin Zhou, Tuo Wang

**Affiliations:** 1 Faculty of Electronic and Information Engineering, Xi'an Jiaotong University, Xi’an, China; 2 Suzhou Academy, Xi'an Jiaotong University, Suzhou, China; Newcastle University, UNITED KINGDOM

## Abstract

Accurate visual tracking is a challenging issue in computer vision. Correlation filter (CF) based methods are sought in visual tracking based on their efficiency and high performance. Nonetheless, CF-based trackers are sensitive to partial occlusion, which may reduce their overall performance and even lead to failure in tracking challenge. In this paper, we presented a very powerful tracker based on the kernelized correlation filter tracker (KCF). Firstly, we employ an intelligent multi-part tracking algorithm to improve the overall capability of correlation filter based tracker, especially in partial-occlusion challenges. Secondly, to cope with the problem of scale variation, we employ an effective scale adaptive scheme, which divided the target into four patches and computed the scale factor by finding the maximum response position of each patch via kernelized correlation filter. With this method, the scale computation was transformed into locating the centers of the patches. Thirdly, because the small deviation of the central function value will bring the problem of location ambiguity. To solve this problem, the new Gaussian kernel functions are introduced in this paper. Experiments on the default 51 video sequences in Visual Tracker Benchmark demonstrate that our proposed tracker provides significant improvement compared with the state-of-art trackers.

## 1. Introduction

Visual object tracking is a crucial research problem in computer vision and has many applications including video surveillance, traffic monitoring, robotics and human computer interface. In the past decade, great improvement has been made by some visual tracking algorithms [1, 2, 3, 4, 5, 6], but visual tracking is still considered as a big challenge in some scenarios such as illumination variation, scale variation, occlusion, deformation and background clutters, etc.

Recently, correlation filter based methods are sought in visual tracking because of their efficiency and high performance. Correlation filters usually generate correlation peaks for each interested patch in one frame while producing low responses to background, which are often used as detectors of expected model. Kernelized Correlation Filter (KCF) tracking has the highest speed while balancing the tracking performance. For a given image, the KCF tracker achieves target tracking by learning the target's appearance by the kernel least squares classifier. However, the KCF tracker does not have the ability to handle the scale problem. Danelljan et al.[7] relieves the scaling issue using feature pyramid and 3-dimensional correlation filter. Yang Li et al.[8] applies a scaling pool to handle scale variations. The above methods have largely solved the scaling problem. Moreover, occlusion is also a tricky problem for these correlation filter based trackers. In general, multi-part tracking scheme can be helpful to gain robustness against partial occlusions. In this respect, Akin et al.[9]proposes a tracker depends on coupled interactions between a global tracker and several part trackers. Jeong et al.[10]applies a naive multi-block scheme based on DSST[7]. These methods can solve partial occlusion to a large extent.

However, negative effects for comprehensive performance of tracker are generated by using sub-part trackers frequently, since sub-part trackers will process part of the target as background during training and detecting. To avoid accumulating negative effects, sub-trackers should only be employed in frames which object is occluded or deformed.

In this paper, we employ an effective spatial distribution to divide target into two sub-parts. To avoid applying sub-trackers frequently, we endue sub-trackers a reliability weight based on the fluctuation of correlation response from globe tracker so that sub-trackers will be chosen only when target is occluded or deformed. We assign different learning rates to different trackers based on the ratio of response values. Moreover, robust scale calculation is a challenging problem in visual tracking. Most existing trackers fail to handle large scale variations in complex videos. To address this issue, this paper proposed a robust and efficient scale-adaptive tracker in tracking-by-detection framework, which divided the target into four patches and computed the scale factor by finding the maximum response position of each patch via kernelized correlation filter. With this method, the scale computation was transformed into locating the centers of the patches. Because the small deviation of the central function value will bring the problem of location ambiguity. To solve this problem, the new Gaussian kernel functions are introduced in this paper.

## 2. Related works

The KCF tracker [11] achieves very excellent results and high-speed performance on Visual Tracker Benchmark [12], despite the ideal and implementation of KCF tracker are very simple. The KCF tracker achieves excellent results and high-speed performance on Visual Tracker Benchmark [10], despite the idea and implementation of KCF tracker are very simple. KCF tracker collects positive and negative samples around the target using the structure of the circulant matrix, to improve the discriminative capability of the track-by-detector tracker. The circulant matrix can be diagonalized with the Discrete Fourier Transform (DFT), enabling fast dot-product instead of expensive Matrix algebra.

The goal of KCF tracker is to find a function that minimizes the squared error over data matrix **X** and their regression target **y**,

$$\underset{w}{\min}\|Xw-y{\|}^{2}+\lambda \|w{\|}^{2}$$ (1)

where the square matrix **X** contains all circulant shifts of the *base sample* **x**, the regression target **y** is Gaussian-shaped, and the λ is a regularization parameter to ensure the generalization performance of the classifier, Eq (1) has the closed-form solution.

$$w={\left(X^{T}X+\lambda I\right)}^{-1}X^{T}y$$ (2)

The circulant matrix **X** has some intriguing properties [16] [11], and the most useful one is that the circulant matrix can be diagonalized by the Discrete Fourier Transform (DFT) as below:

$$X=F^{H}diag\left(\hat{x}\right)F$$ (3)

where **F** is the DFT matrix, and **F**^H^ is the Hermitian transpose. $\hat{x}$ denotes the DFT of **x**, $\hat{x}=F(x)=\sqrt{n}Fx.$

Applying Eq (3) into the solution of linear regression (Eq (2)), we have the solution as below:

$$\hat{w}=\frac{\hat{x}⨀\hat{y}}{{\hat{x}}^{*}⨀\hat{x}+\lambda }$$ (4)

where ${\hat{x}}^{*}$ is the a complex-conjugate of $\hat{x}$. The symbol ⨀ and the fraction denote element-wise product and division respectively.

For detecting the new location of target in the next frame, we can compute the response ***f***(***z***) for all candidate patches **z**, and diagonalize ***f***(***z***) to obtain as below:

$$\hat{f}(z)=\hat{w}⨀\hat{z}$$ (5)

The candidate patch with the maximum response is considered as the new location of target.

## 3. The proposed tracker

In this section, we describe our tracker based on the kernelized correlation filter (KCF) [11]. Firstly, we described the Multi-part tracking tracker, and then the adaptive scale calculation method will be introduced. The selection of Gaussian function is discussed. Moreover, we presented our powerful Multi-part tracking algorithm to improve the correlation filter based trackers.

### 3.1. Multi-part tracking

In visual tracking tasks, partial occlusion is one of the major challenges limiting performance of tracker. Simply, multi-part scheme [13] [14] splits the target into multi-parts and track them independently. When target is partially occluded or deformed, tracker can still locate target rely on the effective sub-part. The high frame rate of KCF also allows multi-part scheme to be applied to real-time tasks. However, the performance of the sub-part tracker does not perform as well as the global tracker in most non-occluded frames, even though sub-part tracker has a higher response value sometimes, since sub-part trackers will process part of the target as background during training and detecting. Therefore, the best method is to use the global tracker when the object is non-occluded, and use a sub-tracker when occlusion occurs.

**Fig 1:**
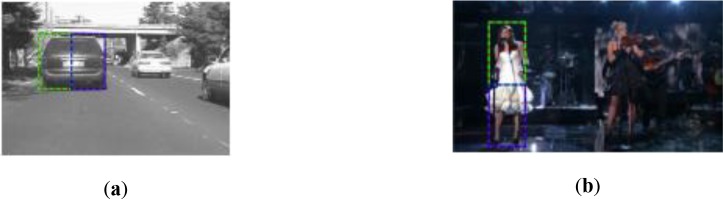
Two spatial distributions based on the ratio of the height and width of the target. The red rectangle represents globe-part, the green and blue rectangles represent two sub-part. (**a**) horizontally aligned object from *car4*; (**b**) vertically aligned object from *single1*;.

In our work, our goal is to develop a multi-part tracker that sub-part trackers and global tracker will take effect in their efficient frames respectively. We employ effective spatial distributions to divide target into two sub-parts, one for the horizontally and one for the vertically aligned object based on the ratio of the height and width of the target. As illustrated in Fig 1.

**Fig 2:**
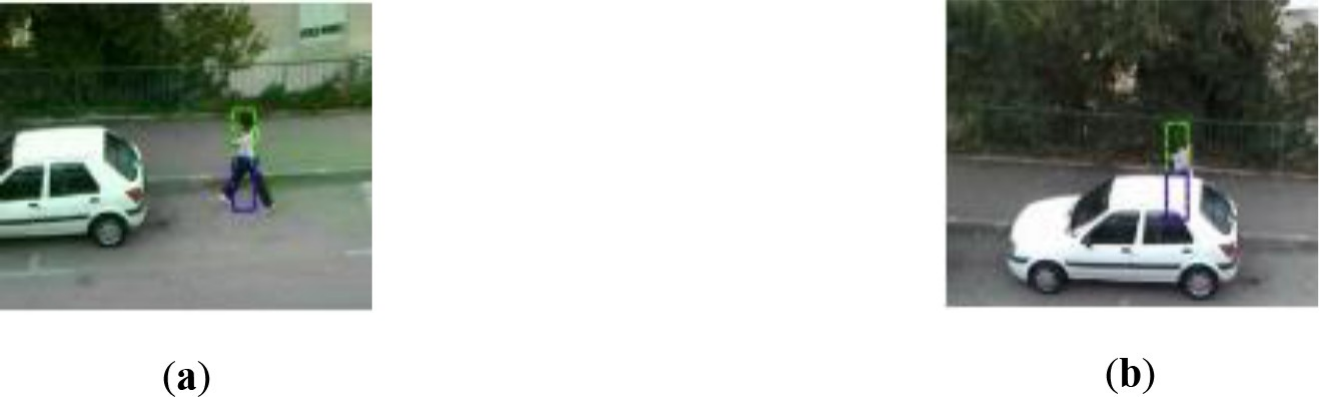
Different optimal tracker for one target in different frame, in (**a**) the globe tracker is better, in (**b**) the green sub-tracker should be selected.

The key in our method is how to select the optimal tracker from both globe and sub-part trackers for different frames, as illustrated in Fig 2. If we simply choose the tracker that has the maximum response, sub-part trackers will be frequently applied to non-occluded frames. Fortunately, when the target is occluded or deformed, the response value of globe tracker will fluctuate significantly relative to frames which the target is non-occluded. Based on above fact, we propose a reliability weight *w* for sub-part trackers. *w* endues multi-part tracker the ability to identify whether the object is occluded or not, and multi-part tracker can select the optimal tracker for different frame itself.

Firstly, we introduce a fluctuation value parameter of global tracker Δ_*t*_.

$$\Delta_{t}=R_{t}^{g}-R_{L}^{g},t>1$$ (6)

for the *1-th* frame of tracking, Δ_1_ is set as 0, the $R_{t}^{g}$ is the globe tracker’s response value of current frame and $R_{L}^{g}$ is the response value of the global tracker which was selected as the optimal tracker for the last time, they can be obtained by Eq (5). The parameter indicates the change of response value after the object is occluded or deformed. The smaller the parameter, the greater the occluded area of the object, that means the globe tracker’s reliability is reduced.

To avoid sub-part trackers are selected as the optimal tracker in non-occluded frames, we assign a reliability weight to response value of sub-part trackers. The reliability weight at the *t*-th frame is defined as:

$$w_{t}=\frac{1+e^{-\eta }}{1+e^{\theta \Delta_{t}}}$$ (7)

where *η* and *θ* are the reliability and sensitivity parameter respectively, in our experiments, *η* sets as 0.4 and *θ* = 1. The reliability weight reduces the probability that the sub- tracker is selected as the optimal one unless the weight is less than -0.4, and it imply the object is likely to be occluded that the reliability weight less than -0.4.

Multi-part tracker can choose the optimal tracker using Eq (8), and $R_{t}^{si}$ is the response value of *i-th* sub-tracker.

$$\begin{array}{c} R^{*}=\arg\;\max(R_{t}^{1},R_{t}^{2},R_{t}^{3}),\\ R_{t}^{1}=R_{t}^{g};\;R_{t}^{2}=w_{t}R_{t}^{s1};\;R_{t}^{3}=w_{t}R_{t}^{s2} \end{array}$$ (8)

If the optimal tracker is globe tracker, the new position can be obtained directly. If one of sub trackers is selected as optimal tracker, the new position can get by shifting in correspondence to the previous center coordinates.

### 3.2. Subsection scale calculation method

Assume the location of the target center in the *t*−1 frame is *p*_*t*−1_, the target scale is *w*_*t*−1_×*h*_*t*−1_. In the *t*−1 frame,take *p*_*t−*1_ as the center,the image block ***z***_***t*−1**_ with size *βw*_*t*−1_×*βh*_*t*−1_ is selected to update the appearance template $\hat{x}$ and coefficient $\hat{\alpha }$,

$$\left\{ \begin{array}{c} {\hat{x}}_{t-1}=(1-\eta ){\hat{x}}_{t-2}+\eta z_{t-1}\\ {\hat{\alpha }}_{t-1}=(1-\eta ){\hat{\alpha }}_{t-2}+\eta \alpha_{t-1} \end{array}\right.$$ (9)

where *β* is expansion coefficient, η is learning rate. Coordinate system is constructed with *p*_*t*−1_ as the origin. The image of *w*_*t*−1_×*h*_*t*−1_ is divided into four equal sub-blocks, and the center of each block is (*w*_1_(*t*−1)×*h*_1_(*t*−1)), (*w*_2_(*t*−1)×*h*_2_(*t*−1)), (*w*_3_(*t*−1)×*h*_3_(*t*−1)) and (*w*_4_(*t*−1)×*h*_4_(*t*−1)), Train the respective linear classifiers on the four sub-blocks, the training class of the classifier (1), the update of the template and the coefficients (9).

In the *t*-frame, the target scale calculation process is, first of all, take *p*_*t*−1_ as the center, selected the image block ***z***_***t*0**_ with size *βw*_*t*−1_×*βh*_*t*−1_. Calculate the maximum response position *p*_*t*_ is the current frame target center location. Then take *p*_*t*_ as the center, selected the image block ***z***_***t*1**_ with size *w*_*t*−1_×*h*_*t*−1_. Coordinate system is constructed with *p*_*t*_ as the origin. Two axes divide image block *w*_*t*−1_×*h*_*t*−1_ into four sub-blocks. Using the classifier trained on the four sub-blocks to find the position with the largest response on the sub-block (*w*_1_(*t*)×*h*_1_(*t*)), (*w*_2_(*t*)×*h*_2_(*t*)), (*w*_3_(*t*)×*h*_3_(*t*)) and (*w*_4_(*t*)×*h*_4_(*t*)), then, the scaling factor *γ*_*t*_ can be given by the relative change of the center position in *w* and *h* dimensions[15]

$$\gamma_{t}=\sqrt{\left(\frac{\sum_{j=1}^{4}\left|w_{j}(t)\right|}{\sum_{i=1}^{4}\left|w_{i}(t-1)\right|}).(\frac{\sum_{j=1}^{4}\left|h_{j}(t)\right|}{\sum_{i=1}^{4}\left|h_{i}(t-1)\right|}\right)}$$ (10)

After calculating the scaling factor *γ*_*t*_, in order to reduce the influence of noise on scale calculation and increase its robustness, moving average (MA) is used to calculate the target scale. Assuming that the moving average parameter is T, the moving average of the expansion coefficient is

$$\rho_{t}=\frac{1}{T}\sum_{i=0}^{T-1}\gamma_{t-i}$$ (11)

In particular, when T = 1 in Eq (11), the moving average degenerates to *ρ*_*t*_ = *γ*_*t*_.

Then, the target scale in the t−th frame is

$$\left\{ \begin{array}{c} w_{t}=\rho_{t-i}w_{t-1}=w_{1}\prod_{i=2}^{t}\rho_{i}\\ h_{t}=\rho_{t}h_{t-1}=h_{1}\prod_{i=2}^{t}\rho_{i} \end{array}\right.$$ (12)

Where *w*_1_ and *h*_1_ were initial frame target sacle.

After calculating the target scale in the t−th frame, take *p*_*t*_ as the center, selected the image block ***z***_***t***_ with size *βw*_*t*_×*βh*_*t*_ to update the appearance template $\hat{x}$ and coefficient $\hat{\alpha }$. At the same time, the *w*_*t*_×*h*_*t*_ target area is divided into four sub-blocks, and the coefficients of the sub-block center, the sub-block template and the classifier on the sub-block are updated.

### 3.3. Selection of Gaussian kernel function

In the tracking algorithm, the objective function generally uses a Gaussian function,

$$y(m,n)=exp(-{\left|p-p_{0}\right|}^{2}/2\sigma^{2})$$ (13)

Where *σ* is constant, *p* = (*m*,*n*), *p*_0_ = (*m*_0_,*n*_0_) is the target center position.

$$|p-p_{0}|=\sqrt{{\left(m-m_{0}\right)}^{2}+{\left(n-n_{0}\right)}^{2}}$$ (14)

Since the partial derivative of the Gaussian function at *p*_0_ = (*m*_0_,*n*_0_) is zero, which is

$$\frac{\partial y}{\partial m}{|}_{p=p_{0}}=\frac{\partial y}{\partial n}{|}_{p=p_{0}}=0$$ (15)

The above equation shows that the deviation of the function value of the objective function near *p*_0_ = (*m*_0_,*n*_0_) is small, and the target position in the tracking process is determined by the maximum response position. Therefore, the small deviation of the central function value will bring the problem of location ambiguity. To solve this problem, the following Gaussian kernel functions are introduced in this paper.

$$\hat{y}(m,n)=exp(-|p-p_{0}|/2\theta )$$ (16)

Where θ>0 is constant. The partial derivative of the function shown in Eq (16)

$$\left\{ \begin{array}{c} \frac{\partial \hat{y}}{\partial m}=-\frac{m-m_{0}}{2\theta |p-p_{0}|}exp(-|p-p_{0}|/2\theta )\\ \frac{\partial \hat{y}}{\partial n}=-\frac{n-n_{0}}{2\theta |p-p_{0}|}exp(-|p-p_{0}|/2\theta ) \end{array}\right.$$ (17)

In particular, the partial derivative at *p*_0_ = (*m*_0_,*n*_0_),

$$\left\{ \begin{array}{c} \frac{\partial \hat{y}}{\partial m}{|}_{m=m_{0}^{+},n=n_{0}}=-\frac{1}{2\theta }\\ \frac{\partial \hat{y}}{\partial n}{|}_{n=n_{0}^{+},m=m_{0}}=-\frac{1}{2\theta } \end{array}\right.\;\left\{ \begin{array}{c} \frac{\partial \hat{y}}{\partial m}{|}_{m=m_{0}^{-},n=n_{0}}=\frac{1}{2\theta }\\ \frac{\partial \hat{y}}{\partial n}{|}_{n=n_{0}^{-},m=m_{0}}=\frac{1}{2\theta } \end{array}\right.$$ (18)

Where $m=m_{0}^{+}$, $n=n_{0}^{+}$ right partial derivative, $m=m_{0}^{-}$, $n=n_{0}^{-}$ left partial derivative.

Eq (18) shows that the left and right partial derivatives of the Gaussian kernel function at *p*_0_ = (*m*_0_,*n*_0_) are not equal, so the partial derivatives at *p*_0_ = (*m*_0_,*n*_0_) do not exist, but both the left and right partial derivatives exist and are constant, which means that the deviation of the target function near *p*_0_ = (*m*_0_,*n*_0_) is large, which is beneficial to the accurate positioning of the target center during the tracking process.

## 4. Experiments

In this section, we first introduce the experimental setup and methodology. Moreover, to evaluate the performance of the proposed Multi-part and Scale Adaptive Tracker (MSAT), we implemented our method to compare with s correlation filter based trackers and other state-of-art trackers on the default 51 video sequence in Visual Tracker Benchmark [12].

### 4.1. Experimental setup and methodology

The proposed tracker is implemented in MATLAB R2014a version. All the experiments are conducted on an Intel Xeon(R) E3-1226 V3 CPU (3.30 GHz) PC with 16GB RAM. The HoG cell size is 4×4 and the number of bin is 9. The padding windows is 2.5 times of target object, and learning rate parameter γ is set to 0.015. The *σ* used in Gaussian kernel is set to 0.5.

We select two quantitative evaluation criteria. The first one is mean overlap precision (OP), OP calculates the percentage of frames in sequences where the intersection-over-union (IOU) overlap exceeds a given threshold of 0.5. The second criteria is the area under curve (AUC), which is computed from the average of the success rates corresponding to the sampled overlap thresholds from 0 to 1.

**Fig 3:**
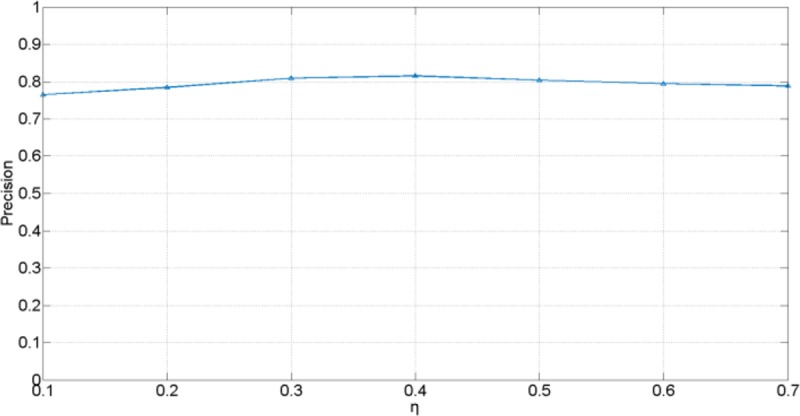
Evaluation of *η* based on precision.

We have tested the performance of the proposed method with different values of the reliability parameter *η*, as shown in Fig 3, the *η* is set from 0.1 to 0.7. The smaller the *η*, the higher the probability that sub-tracker is selected as the optimal tracker. Frequently choosing sub-tracker will reduce performance of the proposed method. On the contrary, assigning *η* too large value is equivalent to using only the global tracker.

To evaluate the comprehensive performance of the proposed approach, we first run seven Correlation Filter-based trackers, and then make comparison with other State-of-art trackers on the default 51 video sequences in Visual Tracker Benchmark [12].

### 4.2. Comparison to correlation filter based trackers

**Fig 4:**
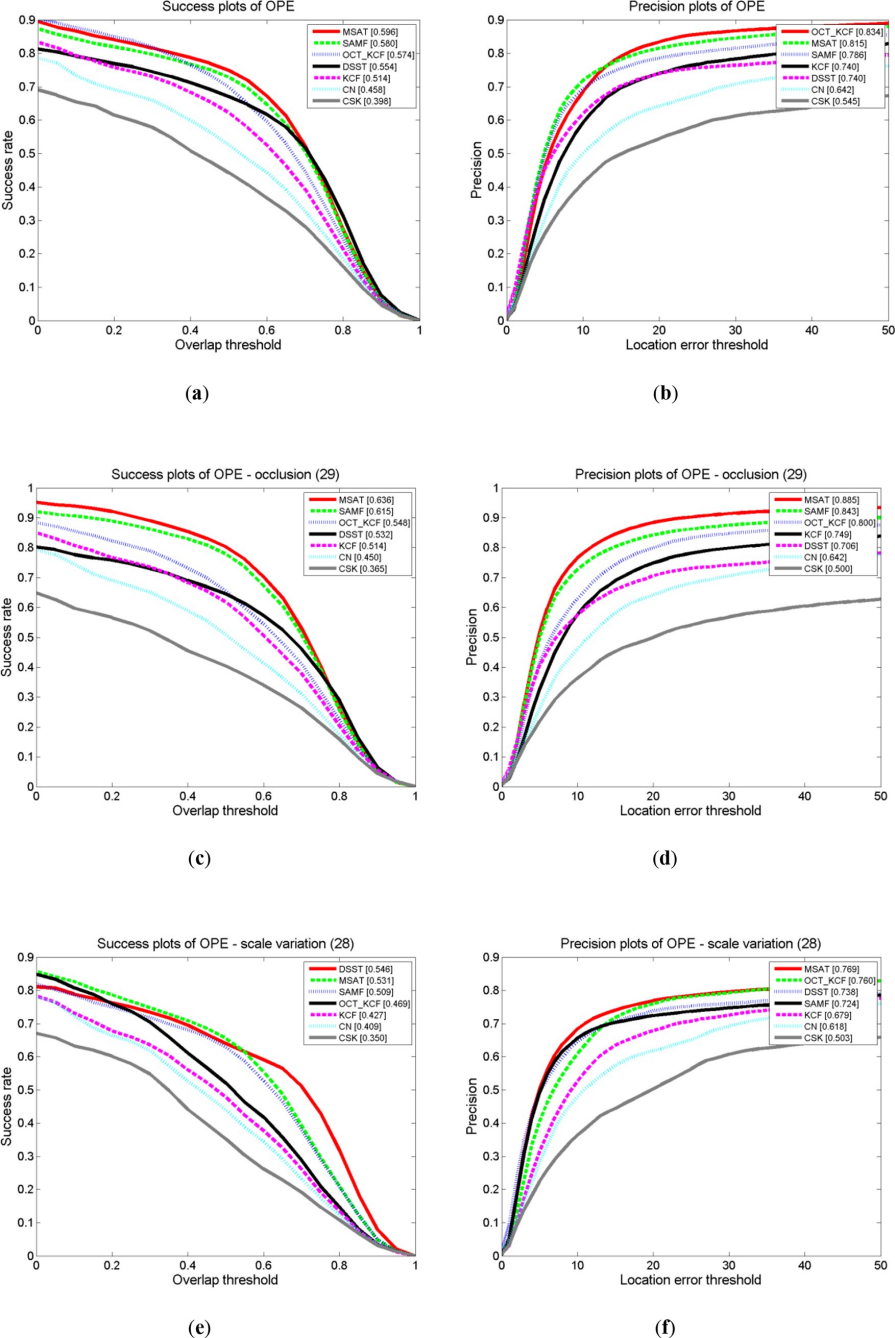
Success plots and precision plots over the default 51 video sequence in visual tracker benchmark [10] for seven kernel correlation filter based trackers. (**a**)- (**f**) indicate the AUC and OP of overall, occlusion and scale variation, respectively.

**Fig 5:**
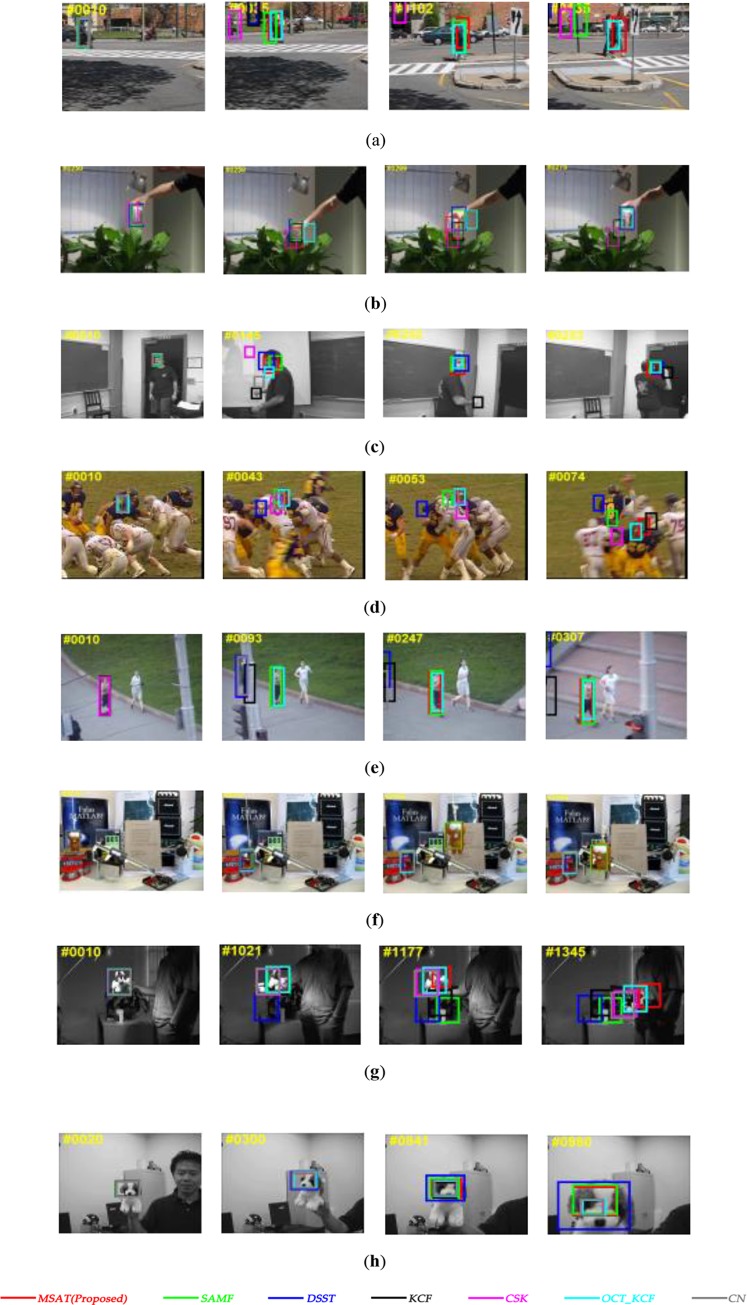
Comparison of our tracker with other kernel correlation filter based trackers[8,9,19,20] in challenging situations. (**a**) couple; (**b**) coke; (**c**) freeman1; (**d**) football1; (**e**) jogging1; (**f**) lemming; (**g**) Sylvester; (**h**) dog1.

To indicate the performance improvements of our approach with multi-part and scale adaptive scheme, we compare our MSAT tracker with the recent correlation filter based trackers that include CSK[16], KCF[11], DSST[7], SAMF[17], OCT_KCF[18],CN[19] on the OTB dataset. All of these trackers are the use of circulant matrix or kernelized correlation filters. Fig 4 shows that mean OP and AUC score of overall, occlusion and scale variation for these trackers. Table 1 summarizes overall comprehensive evaluation for seven trackers. And Fig 5 compare these trackers in challenging situations.

**Table 1:** Overall comprehensive evaluations of kernel correlation filter based trackers.

Tracker	AUC	Mean OP	Speed(FPS)
MSAT(Proposed)	0.596	0.815	9.6
SAMF[17]	0.580	0.786	18
OCT_KCF[17]	0.574	0.834	49
DSST[7]	0.554	0.740	38
KCF[11]	0.514	0.740	219
CN[19]	0.458	0.642	146
CSK[16]	0.398	0.545	294

It is apparent from success plots of Fig 4 that our MSAT tracker has better performance than the other correlation filters based trackers. We also observe from the result that our Multi-part scheme brought high OP and AUC scores in the occlusion challenge, and our tracker is the unique tracker that solves partial occlusion problem in Fig 5(B). Additionally, the results from our experiment shows that trackers(MSAT, SAMF, DSST) explicitly used scale adaptive strategy address the scale change problem have an advantage in the experiments.

The features are essentially significant to the visual object tracking tasks. CSK only employs the raw pixel, whose rank is the lowest one among the correlation filter based trackers. CN uses both raw pixel and color-naming as features, and realizes a lot of improvement upon CSK. Trackers(MSAT, SAMF) with HoG and color-naming features outperform KCF which only employs the HoG feature.

In the precision plots, the OCT_KCF[12] has the highest OP score. Because that the OCT_KCF models the distribution of correlation response in a Bayesian optimization framework to alleviate the drifting problem, making the position in each frame more accurate. In Fig 5(H), the performance of our tracker is inferior to DSST [7] which uses 33 different scales for tracking, but this scale strategy of DSST brings larger cost of computational time.

Table 1 indicates that our tracker has the best overall comprehensive evaluation in seven kernel correlation filter based trackers. Comparing to KCF [11], the MSAT tracker gets a 10.1% and 16% improvement for OP score and AUC score respectively. The result also demonstrates that MSAT promotes the performance of the SAMF [11] which use the same features and scale strategy as our tracker, especially in occlusion challenge. Our proposed MSAT tracker runs at about 10 fps, which is still within real time range.

### 4.3. Comparison with the state-of-art trackers

In our next experiment, we have compared our approach and KCF [11] with 29 different state-of-the-art trackers which reported in the benchmark experiment in [12] on the OTB dataset.

**Fig 6:**
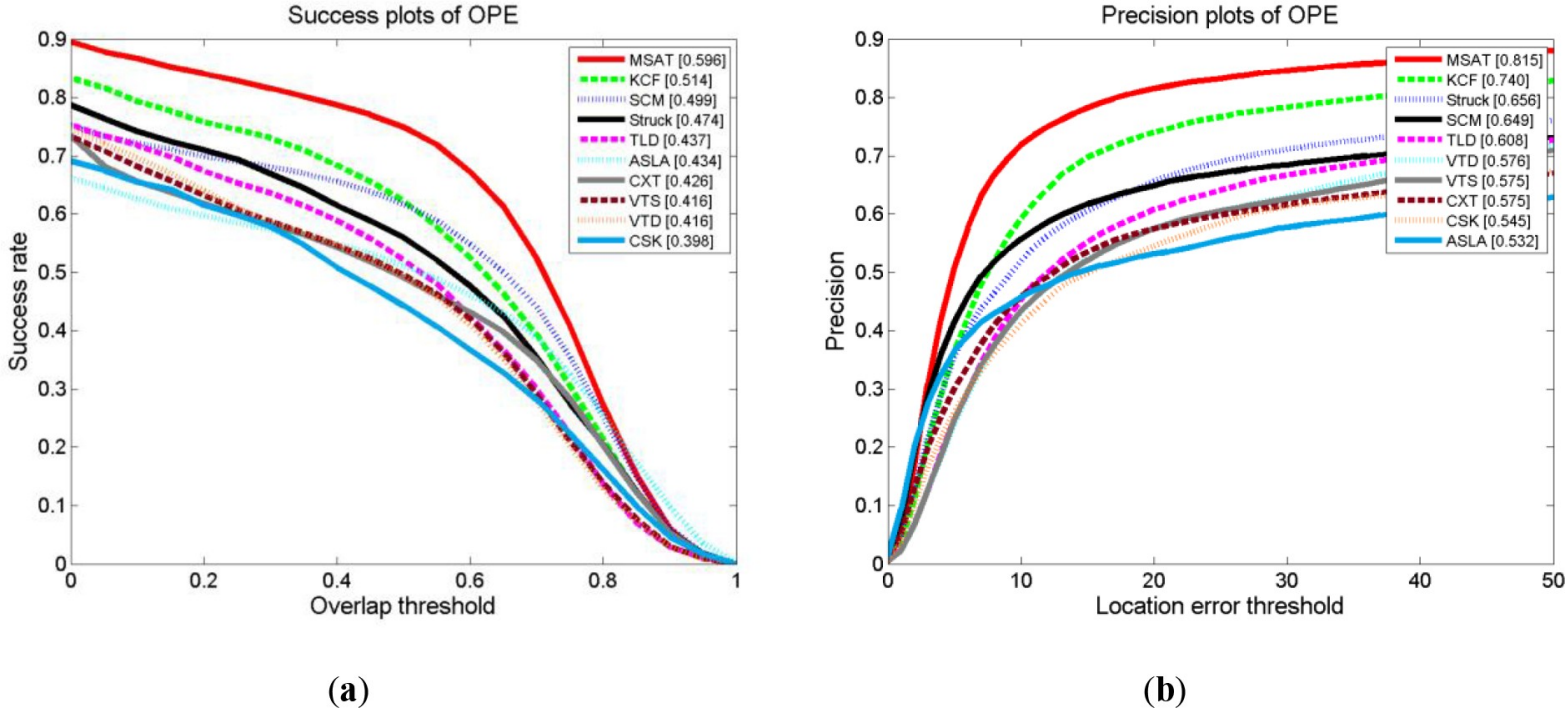
Success plots (**a**) and precision plots (**b**) of proposed tracker against the top nine performing state-of-art tracker[1,2,3,4,5,11,21,17,7,6] on the default 51 video sequence in Visual Tracker Benchmark[12].

Fig 6 presents the overall scores of proposed tracker against the top nine performing state-of-art trackers on the default 51 video sequence in Visual Tracker Benchmark [12]. Correlation Filter Based Trackers (MSAT, KCF, CSK) have the performance with advantage against other State-of-art Trackers. The trackers with HoG feature (MSAT, KCF) achieved an overwhelming performance compared against SCM [4] and Struck [1] in both success and precision plots. The top nine performing state-of-art trackers obtain mean AUC score of 0.446, compared to 0.596 for our MSAT tracker, which is a great improvement for the visual object trackers.

Table 2 shows the mean OP score on the Visual Tracker Benchmark dataset and its challenging sub-categories for the top ten tracking algorithms. Impressively, our MSAT tracker obtains 7 the best and 2 the second best score in 9 sub-categories tasks. The promising result suggests that our tracker with Multi-part and scale adaptive scheme is more effective in the visual tracking challenge.

**Table 2:** Mean OP score on the visual tracker benchmark dataset and its challenging sub-categories for the top ten tracking algorithms[1,2,3,4,5,21,11,8,7,6]. Red and blue label mean the best and second scores, respectively.

Tracker	All	IV	SV	OCC	DEF	MB	FM	IPR	OPR	BC
MSAT	0.815	0.731	0.769	0.885	0.835	0.649	0.694	0.757	0.806	0.729
KCF[11]	0.740	0.728	0.679	0.749	0.740	0.650	0.602	0.725	0.729	0.753
Struck[1]	0.656	0.558	0.639	0.564	0.521	0.551	0.604	0.617	0.597	0.585
SCM[4]	0.649	0.594	0.672	0.640	0.586	0.339	0.333	0.597	0.618	0.578
TLD[5]	0.608	0.537	0.606	0.563	0.512	0.518	0.551	0.584	0.596	0.428
VTD[3]	0.576	0.557	0.597	0.545	0.501	0.375	0.352	0.599	0.620	0.571
VTS[2]	0.575	0.573	0.582	0.534	0.487	0.375	0.353	0.579	0.604	0.578
CXT[8]	0.575	0.501	0.550	0.491	0.422	0.509	0.515	0.610	0.574	0.443
CSK[21]	0.545	0.481	0.503	0.500	0.476	0.342	0.381	0.547	0.540	0.585
ASLA[6]	0.532	0.517	0.552	0.512	0.401	0.352	0.386	0.526	0.519	0.503

## 5. Conclusions

This paper present a very powerful tracker based on the kernelized correlation filter. It proposes a multi-part tracking algorithm to improve the overall capability of correlation filter based tracker, especially in partial-occlusion challenges. By using a reliability weight, we endue multi-part tracking algorithm the ability to select the optimal tracker for different frame itself. Moreover, this paper proposed a robust and efficient scale-adaptive tracker in tracking-by-detection framework, which divided the target into four patches and computed the scale factor by finding the maximum response position of each patch via kernelized correlation filter. With this method, the scale computation was transformed into locating the centers of the patches. In order to solve the problem of location ambiguity, a new Gaussian kernel functions are introduced in this paper. Our proposed MSAT tracker runs at about 10 fps, which is still within real time range. Extensive experiments have been implemented to demonstrate the validity of our proposed tracker.
